# Prevalence of Swine Gastrointestinal Parasites in Nyagatare District, Rwanda

**DOI:** 10.1155/2020/8814136

**Published:** 2020-12-02

**Authors:** M. Tumusiime, P. Ntampaka, F. Niragire, T. Sindikubwabo, F. Habineza

**Affiliations:** ^1^Department of Veterinary Medicine, University of Rwanda, P. O. Box 57, Nyagatare, Rwanda; ^2^Department of Applied Statistics, University of Rwanda, P.O. Box 1514, Kigali, Rwanda

## Abstract

While pig farming has been growing rapidly in Rwanda, its potential contribution to the prevalence of zoonotic infections is not well known. Pig production is usually affected by gastrointestinal parasites, some of which are zoonotic and can threaten human health. The knowledge about the status of such infections is essential for policy decisions and interventions. We aimed to investigate the prevalence of swine gastrointestinal parasites in Nyagatare district, Rwanda. A cross-sectional study involved collecting 104 faecal samples from apparently healthy pigs. The floatation technique was used to identify the parasites and frequency distribution analysis, and Pearson chi-square tests of association were conducted for this study data. Overall, the prevalence of swine gastrointestinal parasites was 84.6%, and the predominant species were *Strongyle*-type helminths representing 70.2%, followed by coccidia (55.8%), *Strongyloides ransomi* (39.4%), and *Ascaris suum* (10.6%). Of all parasitized pigs (*n* = 88), 84.1% developed coinfections involving 2, 3, or 4 different parasite species. The results showed a statistically significant correlation between the location of pigs and parasitic infections and that some prevalent parasites are zoonotic. Interventions among pig farmers in Nyagatare should aim to improve awareness and to provide information on the negative impacts of swine gastrointestinal parasites on pig production and human health.

## 1. Introduction

In 2018, pork production represented 21% of meat production in Rwanda and pig population was estimated to be 1,330,461 [1]. Between 2013 and 2018, pig population in Rwanda increased by 76% [2]. The government of Rwanda expects to increase pork production from 19,945 to 67,676 metric tons of pork between 2017 and 2021 [3].

Among critical obstacles to such a country target are parasitic infections that can cause significant economic losses to pig production [4]. Swine parasites can live in different body parts of the hosts including the lungs (*Metastrongylus* spp.), kidney (*Stephanurus dentatus*), muscle (*Cysticercus cellulosae*, *Toxoplasma gondii*), and gastrointestinal tract [5, 6]. The parasites of gastrointestinal tract include protozoa such as coccidia (*Cystoisospora* spp. and *Eimeria* spp.) and *Entamoeba* spp., as well as helminths. The latter include, among others, *Strongy*le*-*type helminths (*Oesophagostomum* spp., *Hyostrongylus rubidus*), *Strongyloides* spp., *Ascaris* spp., *Trichuris* spp., and *Fasciolopsis* spp. [7–9]. Pigs infected with gastrointestinal parasites grow slowly, can produce small litter size, and can die in case of heavy infection [10]. In addition, some parasites of pigs are responsible for zoonotic infections. These include among others *Toxoplasma gondii*, *Trichinella spiralis*, *Taenia solium*, *Sarcocystis suihominis*, *Ascaris suum*, *Entamoeba polecki*, and *Fasciolopsis buski* [5, 11–14]. There is a wide range of transmission routes through which pigs can contract parasitic infections, among others, oral, colostral, and percutaneous. The oral transmission can occur through ingesting infective larva (*Strongyle*-type worms), embryonated eggs (*Trichuris suis*, *Ascaris suum*), items contaminated with metacercaria (*Fasciolopsis* spp.), cysts (*Entamoeba* spp.), or sporulated oocytes (coccidia) [6, 14, 15]. The transmission of *Strongyloides ransomi* involves the ingestion or suckling of items or colostrum contaminated with infective larvae as well as larval penetration of the host skin [6, 10].

During infection, gastrointestinal parasites can irritate intestinal mucosa and negatively affect the digestion and absorption of nutrients [8]. For instance, slight infection with *Ascaris suum* can decrease food intake and daily weight gain in infected animals [9, 16]. Different factors can influence the prevalence of swine parasites including the system of management (intensive, semi-intensive, and extensive), on farm hygiene, geographical location, and antiparasitic prophylactic treatment such as deworming [5, 6, 17, 18]. There are no published research works on gastrointestinal parasitoses of pigs in Rwanda and the information on such infections is scarce. In addition, studies carried out in other places reported the prevalence of gastrointestinal parasitoses in pigs that varies between 82% and 97% [19, 20]. This study determined the prevalence of swine gastrointestinal parasites in Nyagatare district, Rwanda. The study results are expected to provide valuable information about parasitic infections of pigs. The information can help in prioritising interventions to improve productivity of pig farming and to minimize the risk of transmitting zoonotic parasites to livestock and humans.

## 2. Materials and Methods

### 2.1. Study Area

The study was carried out in Nyagatare district, one of the 7 administrative districts of Eastern province, Rwanda, between April and May 2019. Rwanda is in central East Africa, and it is administratively divided into 30 districts. Nyagatare district is divided into 14 sectors and 106 cells. It borders two districts, namely, Gatsibo (South) and Gicumbi (West), and two countries, namely, Uganda in the North and Tanzania in the East [21]. In 2012, the whole population of pigs in the Eastern province was estimated to be 104,000 and accounted for 10.5% of the pig population in Rwanda [1, 22].

**Figure 1:**
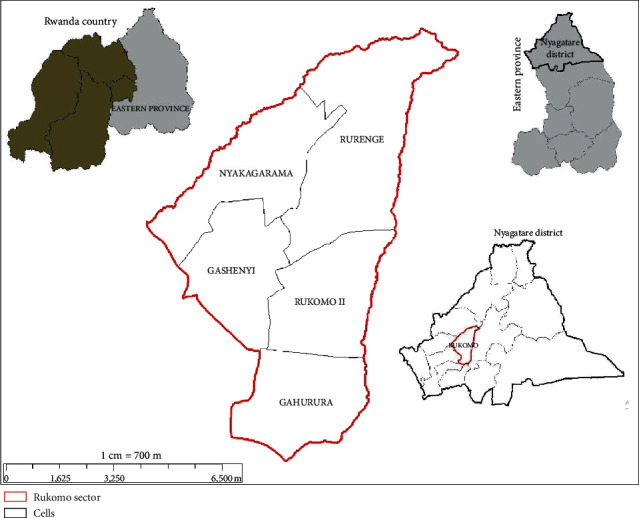
Map of Nyagatare district and the study area.

In this study, Nyagatare district was purposively selected because it is an area dedicated to farming. It is a district with the highest cattle population in Rwanda and where pig production has been gaining attention. In addition, research in Nyagatare has mainly focused on cattle and small ruminants and has overlooked the importance of the fast-growing pig population in the district.  Figure 1 shows the map of Nyagatare district and the study area. The figure shows Rukomo administrative sector (red limit) and four of its administrative cells (gray), namely, Rurenge, Nyakagarama, Gashenyi, and Rukomo II. These cells are the sampling sites in the present study.

### 2.2. Study Design and Sampling Procedure

The present cross-sectional study involved collecting faecal samples from apparently healthy pigs younger than 1 year old and kept under semi-intensive system. In the first stage, cluster sampling was used to randomly select Rukomo administrative sector as a cluster in Nyagatare district. In the second stage, the study pigs were randomly selected across four administrative cells in Rukomo sector, namely, Gashenyi, Nyakagarama, Rukomo II, and Rurenge. Records showed that the pig population in the Rukomo sector was around 2500 in 2018. There were few pigs in Gahururu administrative cell, and thus, it was not considered in this study. For calculation of this study sample size, the prevalence of swine parasitoses in Rwanda was assumed to be 50% [23], as there was no prior knowledge about it.

With a level of precision of 10% and a 95% level of confidence, the minimum size *n* of a representative sample was computed using Cochran's sample size formula for proportions, given as Equation (1) [23].

(1) $$\begin{array}{c} n=\frac{\left(Z^{2}\cdot p\left(1-p\right)\right)/e^{2}}{1+\left(\left(Z^{2}\cdot p\left(1-p\right)\right)/e^{2}N\right)}=96, \end{array}$$

where *N* is the pig population size.

The population of Rwanda is usually responsive to health research studies, but they are not familiar with studies on domestic animals; thus, we increased the computed sample size by 10% to compensate for possible nonresponse [24]. Therefore, this study targeted a total sample size of 106 pigs of whom 104 were successfully sampled.

### 2.3. Collection of Samples

Prior to collecting the samples, pig farmers were briefed about the purpose of the study. Faecal samples were collected from pigs owned by the farmers who authorised sampling of their animals.

The samples were collected from rectum using gloved fingers, put in a stool container, and transported in a cool box. In the laboratory, the samples were kept refrigerated and analysed the following day. The analysis was performed at the laboratory of the School of Veterinary Medicine of the University of Rwanda. The samples were processed by floatation method for nematode eggs and protozoal ova and cysts while sedimentation technique was used for trematode eggs. The preparation of floatation fluid involved dissolving 400 g of sodium chloride in a litre of tap water, that is, the fluid had a specific gravity of 1.200. The samples were prepared by mixing 3 g of faeces in 50 ml of floatation fluid [25]. The suspension was filtered through a tea strainer into a beaker and then transferred to a test tube until a positive meniscus was reached. Then, a cover slip was put on top of the test tube for 10 minutes after which it was removed and mounted on a microscope slide. To perform the sedimentation, 3 g of faeces was dissolved into 100 ml of water in a beaker, and then, the suspension was filtered using a tea strainer into another beaker and left to sediment for 10 minutes. After 10 minutes, the supernatant was removed carefully leaving the sediment, and then, 100 ml of water was added to the sediment, and the same process was repeated three times. At the end of the third time, the sediment was put on a microscopic slide and examined under a microscope. Based on their size and shape, the eggs or cysts of gastrointestinal parasites were identified under microscope at 10x magnification [26].

### 2.4. Data Analysis

The sample test results together with respective information on the location (cell) of the sampled pigs were entered into IBM Statistical Package for Social Sciences (SPSS) version 23. Frequency distribution analysis was performed, and the overall prevalence was calculated. To compute the prevalence, the number of pigs infected with gastrointestinal parasites was divided by the total number of sampled pigs multiplied by hundred.

Any association between the prevalence of gastrointestinal parasites and sampling sites was assessed using Pearson chi-square test of independence [27]. The results were considered significant at 5% level of significance.

## 3. Results

The data were successfully collected from 104 pigs younger than 1 year old and kept under semi-intensive system. Overall estimate of the prevalence of gastrointestinal parasites in study pigs is presented in Table 1.

The results in Table 1 show that the overall prevalence of swine gastrointestinal parasites was 84.6%. Administrative cell-level-specific prevalence of different gastrointestinal parasites and tests of association with sampling sites are shown (Table 2).

The results in Table 2 indicate that there is a statistically significant association between the prevalence of *Strongyle*-type worms and coccidia with the location of the pigs (sampling sites).

The relationship between sampling sites and the number of parasite species detected per study pig is indicated in Table 3.

**Figure 2:**
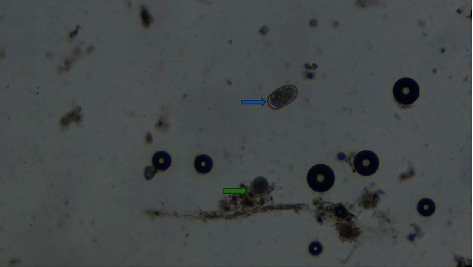
*Strongyle*-egg type (blue arrow) and *Entameoba* cyst (green arrow).

**Figure 3:**
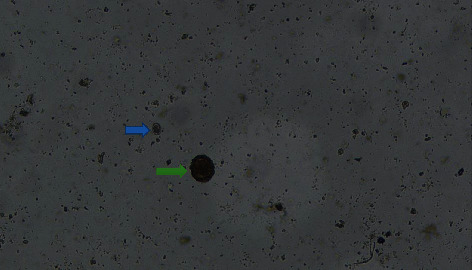
*Ascaris suum* (green allow) and coccidian cyst (blue arrow).

**Figure 4:**
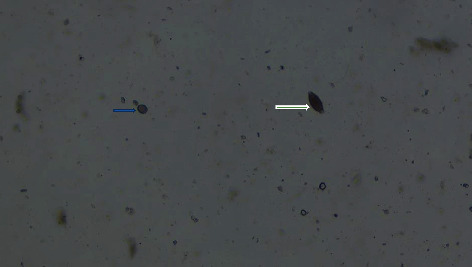
Coccidian cyst (blue arrow) and *Trichuris suum* (white arrow).

**Figure 5:**
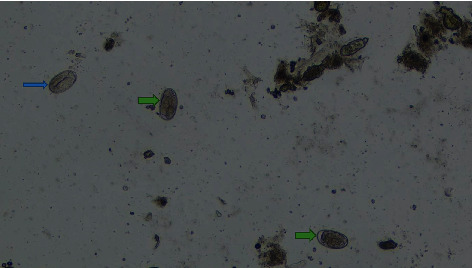
*Strongyle*-egg types (arrows green) and *Strongyloides ransomi* (blue arrow).

**Figure 6:**
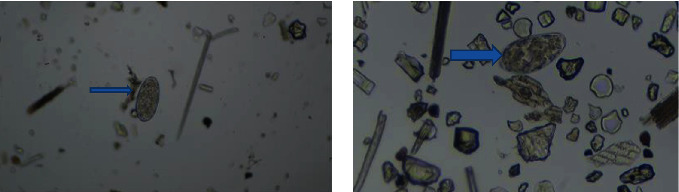
*Fasciolopsis* spp. (blue arrows).

Of all infected pigs, 84.1% developed coinfection, and majority (53.4%) were infected with two types of parasites. The results in Table 3 show that the number of parasite categories is not significantly influenced by the geographical location of the pigs across the sampling sites. Figures 2–6 illustrate eggs and cysts of the parasites.

## 4. Discussion

This study aimed at investigating gastrointestinal parasites in pigs in Nyagatare district, and the findings can help in prioritising interventions for improving productivity of pig farming and minimizing the risk of transmitting zoonotic parasites to livestock and humans. The overall prevalence of gastrointestinal parasites was 84.6%, and three of the identified types of parasites are zoonotic, including *Ascaris suum*, *Fasciolopsis* spp., and *Entamoeba* spp. Our findings also show that the prevalence of *Strongyle*-type helminths and coccidia in pigs in Nyagatare was significantly associated with the geographical location of the pigs.

The prevalence rates for some parasites were very high, while they were relatively lower for others compared to previous studies conducted in different countries. For instance, the study overall prevalence of gastrointestinal parasites reported in this study was lower than 91%, 93%, and 97% reported in studies conducted in Ghana, Burkina Faso, and China, respectively [20, 28, 29]. However, it was higher than 82% reported by Nsoso et al. in Botswana [19]. The prevalence of 70.2% recorded for *Strongyle*-type helminths in the present study was higher than 52% reported by Boes et al. in China [20].

This study prevalence of coccidia (55.8%) was lower than 47.2% and 40.7% reported by Weng et al. in China and Roesel et al. in Uganda, respectively [30, 31]. However, it was higher than that conducted by Karamon et al. in Poland [32] which found that 27.8% of investigated piglets had *Isopsora* (*Cystoisospora*) *suis* while 2.6% had *Eimeria* spp. This study prevalence of 39.4% for *Strongyloides ransomi* was higher than 4.2% reported in Uganda by Roesel et al. [31]. One of the important factors that can explain the difference in prevalence is thought to be the production system. All the pigs investigated in this study were reared under semi-intensive system while pigs investigated by Roesel et al. in Uganda [31] were reared under different production systems including intensive, semi-intensive, and extensive.

Compared to the studies conducted in Uganda and Tanzania [10, 31], the prevalence of *Ascaris suum* was lower in Uganda and higher in Tanzania than in Nyagatare, Rwanda. Apart from *Ascaris suum*, two other types of potential zoonotic parasites, namely, *Fasciolopsis* spp. and *Entamoeba* spp., were also identified.

*Fasciolopsis buski* is a zoonotic parasite, and it is prevalent in pigs in different Asian countries and has been reported in Africa, for instance, in Nigeria [33, 34]. There are no published studies on fasciolopsis in Rwanda, but it is a food-borne infection, and humans develop it after they eat raw aquatic vegetation or food plants contaminated with metacercariae [33]. Pigs can develop amoebiasis due to *Entamoeba suis* and *E. polecki*, and the latter one is a zoonotic species [13].

Other common zoonotic parasites include *Taenia solium*, *Trichinella spiralis*, *Toxoplasma gondii*, and *Sarcocystis suihominis* [11, 17]. A study conducted in southern part of Rwanda found that larvae of *Taenia solium* (*Cysticercus cellulosae*) caused 23.3% of epileptic cases in people [35]. There is no information about the prevalence of trichinellosis in humans and pigs in the study area and in Rwanda at large.

Although there are no published studies on toxoplasmosis in animals in Rwanda, a study carried out in pregnant women in Kigali found that 12.2% were positive for *Toxoplasma gondii* and that the occurrence was associated with drinking unclean water and eating undercooked meat [36].

Similar to reports by Nansen and Roepstorff [37] and by Roesel et al. [31], the present study showed that the geographical location of the pigs was significantly associated with the prevalence of *Strongyle-*type helminths and coccidia in pigs in Nyagatare. We found that the rate of mixed infections in pigs suffering from parasitoses represented 84.1%, and it was higher than 7% reported by Atawalna et al. in Ghana [4]. It is more likely that coinfections found in this study would considerably contribute to the reduction of the production and performance of the pigs [10]. Although pig farmers may not be aware due to subclinical infections, economic losses caused by endoparasites are important [17].

## 5. Conclusions

The prevalence of swine gastrointestinal parasites was as high as 84.6%, and the predominant species were s*trongyle*-type helminths, followed by coccidia. The prevalence of gastrointestinal parasites is high and varies according to the location of the pig in Nyagatare, Rwanda. In addition, some detected parasites including *Ascaris suum*, *Fasciolopsis* spp, and *Entameoba* spp. are zoonotic. The intervention is needed to raise awareness among pig farmers about negative impacts on pig farming productivity and on other livestock as well as on human health.

## Figures and Tables

**Table 1:** Overall prevalence of swine gastrointestinal parasites in Nyagatare district.

Results	Number of pigs (*n* = 104)	Percent
Negative	16	15.4
Positive	88	84.6
Total	104	100.0

**Table 2:** Prevalence and test of association of gastrointestinal parasites with study locations (*n* = 104).

Parasite	Frequency				Test of independence
	Site	Sample	Positive cases (%)	Prevalence	*P* value
*Strongyle-*type helminths	Rukomo II	20	17 (23.3%)	85%	0.002
	Gashenyi	39	27 (37.0%)	69.2%	
	Nyakagarama	31	25 (34.2%)	80.6%	
	Rurenge	14	4 (5.5%)	28.6%	
	Total	104	73 (100%)	70.2%	

*Entamoeba* spp.	Rukomo II	20	1 (50%)	5%	0.578
	Gashenyi	39	1 (50%)	2.6%	
	Nyakagarama	31	0	—	
	Rurenge	14	0	—	
	Total	104	2 (100%)	1.9%	

*Coccidia*	Rukomo II	20	13 (22.4%)	65.0%	0.014
	Gashenyi	39	20 (34.5%)	51.3%	
	Nyakagarama	31	22 (37.9%)	71.0%	
	Rurenge	14	3 (5.2%)	21.4%	
	Total	104	58 (100%)	55.8%	

*Strongyloides ransomi*	Rukomo II	20	9 (22.0%)	45.0%	0.237
	Gashenyi	39	19 (46.3%)	48.7%	
	Nyakagarama	31	10 (24.4%)	32.3%	
	Rurenge	14	3 (7.3%)	21.4%	
	Total	104	41 (100%)	39.4%	

*Ascaris suum*	Rukomo II	20	1(9%)	5.0%	0.778
	Gashenyi	39	5 (45.5%)	12.8%	
	Nyakagarama	31	3 (27.3%)	9.7%	
	Rurenge	14	2 (18.2%)	14.3%	
	Total	104	11 (100%)	10.6%	

*Trichuris* spp.	Rukomo II	20	0	—	0.074
	Gashenyi	39	4 (100%)	10.3%	
	Nyakagarama	31	0	—	
	Rurenge	14	0	—	
	Total	104	4 (100%)	3.8%	

*Fasciolopsis* spp.	Rukomo II	20	0	—	0.223
	Gashenyi	39	1 (25%)	2.6%	
	Nyakagarama	31	3 (75%)	9.7%	
	Rurenge	14	0	—	
	Total	4	4 (100%)	3.8%	

**Table 3:** Number of parasite categories detected per infected study pig according to sampling sites (*n* = 88).

Sampling site	Number of parasite categories				Total	Chi-square tests
	One	Two	Three	Four		
Rukomo II	1	12	4	1	18	*X* ^2^ = 9.546, *P* = 0.388
Gashenyi	5	15	10	3	33	
Nyakagarama	5	17	8	0	30	
Rurenge	3	3	1	0	7	
Total	14	47	23	4	88	
Percent	15.9	53.4	26.1	4.5	100.0	

## Data Availability

All relevant data collected and analysed during this study are within the manuscript.
